# A Case Report of Extensive Cerebral Venous Sinus Thrombosis as a Presenting Sign of Relapsing Nephrotic Syndrome

**DOI:** 10.1155/2019/6840240

**Published:** 2019-12-27

**Authors:** Janet K. Lee, Kathleen Murray, Swetha Renati

**Affiliations:** University of South Florida, Department of Neurology, Tampa, FL, USA

## Abstract

Nephrotic syndrome is defined by three characteristic features including proteinuria of >3 g in 24 hours, hypoalbuminemia of less than 3 g/dL, and peripheral edema. Multiple nephropathies can result in nephrotic syndrome. Most commonly, minimal change disease is seen in children under the age of 10, while adults are more commonly found to have membranous nephropathy. Hypercoagulability and thrombotic sequela can be seen in nephrotic syndrome, regardless of underlying etiology, and thrombosis is most commonly seen in deep veins of the lower extremities and renal veins. Our case identifies an adult with previously diagnosed and treated for minimal change disease who presented with weight gain, peripheral edema, foamy urine, headache but no neurologic deficits. The patient was found to have near to complete occlusion of the entire superior sagittal sinus, near complete occlusion of the left transverse and sigmoid sinuses, and nonocclusive thrombus in the right sigmoid sinus. She was treated with heparin and IV steroids then transitioned to warfarin and PO steroids, respectively, with resolution of symptoms. This case report emphasizes on the importance of recognizing CVST as a potential complication of nephrotic syndrome at both initial presentation and relapse.

## 1. Introduction

Nephrotic syndrome is characterized by proteinuria, hypoalbuminemia, edema, and hyperlipidemia. It is associated with hypercoagulability with thrombosis occurring most frequently in the deep veins of lower extremities and renal veins [1]. Cerebral venous sinus thrombosis (CVST) is a rare complication of nephrotic syndrome.

## 2. Case Report

**Figure 1:**
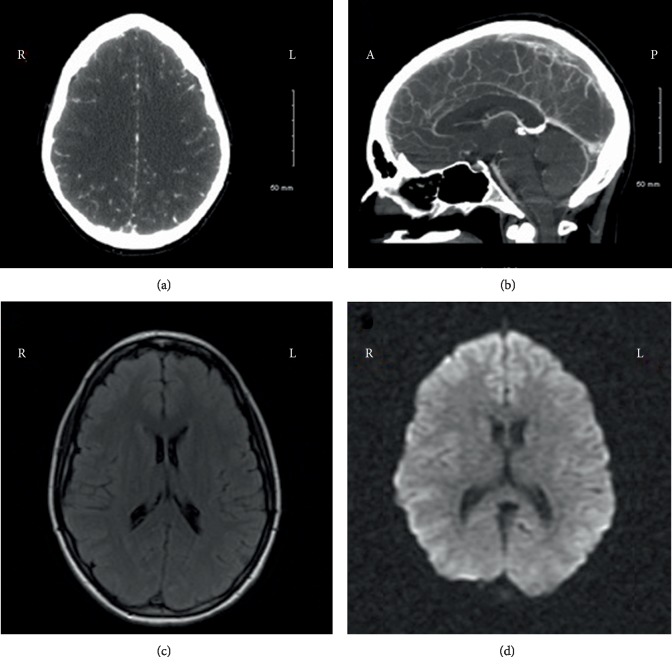
CTV and MRI showing extensive cerebral sinus thrombosis. (a) CT venogram. Axial view. Showing empty delta sign and thrombus in the superior sagittal sinus. (b) CT venogram. Sagittal view. Showing filling defect in the superior sagittal sinus consistent with thrombotic occlusion of superior sagittal sinus. (c) MRI brain T2 Fluid-attenuated Inversion Recovery (FLAIR). Axial view. Showing no parenchymal involvement. (d) MRI brain Diffusion weighted imaging (DWI). Axial view. Showing no acute ischemia or cytotoxic injury.

A female in her early 20s with a recent diagnosis of biopsy proven Minimal Change Disease (MCD) presented with headache, neck pain, photophobia, nausea, and vomiting. Patient was diagnosed with MCD approximately 2 months prior and started on prednisone. During follow-up, proteinuria improved from 7 g/day to 0.5 g/day and hypoalbuminemia improved from 0.5 g/dL to 2.2 g/dL. Given improvement she was started on a taper of the steroids. As the prednisone was being tapered off she started having symptoms of foamy urine, edema, and weight gain. Her headache started 8 days prior to presentation. She was seen at an outside hospital 1-week prior for headache and was treated with sumatriptan and acetaminophen/butalbital/caffeine with only minimal improvement. Her headache continued to worsen with associated blurry vision and photophobia. On examination, vitals were within normal range and the neurologic exam was significant only for bilateral Grade 1 papilledema. CT angiogram of the head with venous phase showed near to complete occlusion of the entire superior sagittal sinus, near complete occlusion of the left transverse and sigmoid sinuses, and nonocclusive thrombus in the right sigmoid sinus (Figure 1). The patient was admitted and a continuous unfractionated heparin infusion was started. She was found to have a relapse of her nephrotic syndrome and was started on intravenous corticosteroids. Albumin levels were low at 1.6 g/dL. However, renal function was within normal limits. No prior thrombotic history was reported and hypercoagulable labs were unrevealing. She was transitioned to warfarin and was treated for nephrotic syndrome with prednisone 1 mg/kg/day. Oral contraceptive was discontinued. Patient's headache improved with no residual neurological deficits.

Following discharge, the patient was continued on high dose prednisone and a weaning trial was attempted but resulted in worsening of nephrotic syndrome symptoms. She was eventually started on mycophenolate and successfully weaned off prednisone. Warfarin was continued for 10 months and discontinued following improvement of her nephrotic syndrome. At the time of anticoagulation discontinuation, her albumin improved to 4.8 g/dL. Neurologically, she remained asymptomatic with no recurrence of symptoms.

## 3. Discussion

Nephrotic syndrome is associated with hypercoagulability. While the mechanism remains poorly understood, it is generally thought to be caused by an imbalance of procoagulant and anticoagulant factors [2]. The risk of thrombotic complications in nephrotic syndrome is highest earlier in the disease course [2] and increases at lower albumin levels with the risk highest in membranous nephropathy. Lionaki et al. found that those with albumin levels <2.8 g/dL have a 2.5-fold increased risk of thromboembolic events [3]. Other risk factors include high proteinuria and age, with adults having a 7-8 fold increase in incidence compared to children [1]. Venous thrombosis is more frequent, and most commonly reported in the deep veins of the lower extremities followed by the renal veins [1]. Patients with nephrotic syndrome also have an increased risk of pulmonary emboli, likely secondary to the increased risk of deep vein thrombosis (DVT). Cerebral venous sinus thrombosis remains a rare or possibly underdiagnosed complication of nephrotic syndrome. In children, only 35 cases were reported from 1980 to 2005 [4]. For adults, the incidence of CVST in the context of nephrotic syndrome and specifically minimal change disease is unknown.

Minimal Change Disease (MCD) causes approximately 90% of cases of nephrotic syndrome in children but is estimated to cause only 10–25% of nephrotic syndrome cases in adults [5]. Compared to children, adults with MCD more commonly present with acute kidney injury and respond less rapidly to steroid therapy [5]. Corticosteroid therapy has been shown to lead to complete remission in over 80% of adults but often requires 12–16 weeks of therapy [5].

In our review of the literature, there are few documented cases of cerebral thrombosis in the context of adult MCD. Only 10 cases involved cerebral venous sinus thrombosis [6–14]. Patients were 20 to 68 years old, with one patient having documented prothrombotic risk factors of OCP use and smoking history. Most patients presented with new onset MCD while at least three documented cases were relapsing. Clinically, patients presented with progressive yet vague neurological complaints. Symptoms included new onset of focal or generalized seizures or signs of increased intracranial pressure including: intractable headache, nausea, vomiting, visual changes, papilledema, or changes in mental status. In the majority of cases with CVST, the superior sagittal sinus was involved followed by transverse sinuses.

Treatment of CVST in the setting of nephrotic syndrome includes the use of corticosteroids and anticoagulation. Anticoagulation with a continuous unfractionated heparin infusion followed by warfarin is preferred. In CVST from a provoked transient risk factor (such as OCP) it is reasonable to continue anticoagulation for 3–6 months based on clinical and imaging improvement [15]. However nephrotic syndrome presents a unique scenario where anticoagulation may need to be continued longer in high risk patients with hypoalbuminemia. Given the increased risk of thrombosis at lower albumin concentrations it has been proposed that prophylactic anticoagulation should be considered in high risk patients with serum albumin <2.0 g/dL [16].

Although rare, our case emphasizes the importance of recognizing CVST as a potential complication of nephrotic syndrome, particularly in patients with prothrombotic risk factors.
